# A Case of Hepatic Abscess, Which Discharged Pus at Three Points

**Published:** 1844-05

**Authors:** Andrew R. Kilpatrick

**Affiliations:** Woodville, Miss.


### WESTERN JOURNAL

OF

### MEDICINE AND SURGERY.

MAY, 1844.

Art. I.—*A Case of Hepatic Abscess, which discharged pus at three points.* By Andrew R. Kilpatrick, M. D., of Woodville, Miss.

In looking over the medical literature of the day one is struck by the number of cases of strange or wonderful disease—of recovery from wounds apparently immedicable, or diseases of the most hopeless character, and a doubt may sometimes arise whether such things can be so. But the physician of extensive experience or reading has been taught that, in our profession,

"Truth is strange,—stranger than fiction."

The following case, if I am not mistaken, will be considered remarkable. If there is another on record precisely like it, I have not been able to find the history of it, and I have looked diligently through all the medical books within my reach. It occurred in the Grand Prairie, St. Landry Parish, Louisiana, in the practice of Dr. James M. Murph, of Washington, near Opelousas.

Stephen Bundick, aged 33 years, large and well formed, a blacksmith by trade, health generally good, but occasionally interrupted by attacks of intermittent fever, and pain in the right hypochondrium, which latter symptom was increased by hard labor. On the 23d of February, 1843, he was attacked by severe pain in the region of the liver, which yielded to venesection and cathartic medicine. The pain returned next day with its former violence; the same remedial agents were again employed, but the bleeding was not carried to a sufficient extent, as the pain was only slightly moderated, and continued, in spite of free catharsis, up to the morning of the 3d of March, when the pain suddenly diminished, leaving a dull, heavy pain, as he styled it. This was regarded as a favorable crisis, and he and his family looked with hope to a speedy restoration of health. The scene, however, soon changed; his extremities became cold, and a clammy perspiration bedewed the body. By employing stimulants and rubefacients a slight degree of warmth was restored, but he still continued to sink, becoming partially comatose, with difficult, laborious respiration; and in the afternoon what was regarded as the ominous death-rattle added to the other symptoms its unwelcome sound. It was at this late stage of the case that Dr. Murph was called in. The hands and feet of the patient were very cold; eyes, though dull, still indicated consciousness and apprehension, as he gazed in his physician's countenance. Pulse small, soft, and thread-like, so much so as to be scarcely perceptible; tongue moist, clean at the tip and edges, brown and fissured in the centre and at the base; dull pain in the right side; slight tenderness in the epigastrium; spleen and liver both considerably enlarged; intestines had been emptied by free purgation. Stimulants were now given freely, and frictions and mustard sinapisms applied to the extremities, followed by vesicatories. Scarified cups to right side of thorax, after which ung. tart. ant. was rubbed on the scarified places, and a large vesicatory was applied to right hypochondrium. He was revived by the stimulants, and by turning him on the right side the mucus was dislodged from the trachea, and his respiration became more easy. Left powders of submur. hydrarg., sulph. quinine and gum camphor, to be administered every three hours and assisted, if necessary, by enèmata.

March 4th. Pulse fuller; skin more natural in warmth and color; blisters drew well; expectoration more easy and free; medicine acted rather too much. Continued same powders with the addition of morphia.

5th, 6th, and 7th. Visited him regularly, and his amendment was progressive and marked. Pustules from ung. tart. ant.; blistered surfaces secreting and bowels soluble; appetite returning. There is still a disinclination to lying on the left side, and occasionlly an obtuse pain in right side. Giving general directions, visits were discontinued. Saw him again on the 27th March, at which time he was expectorating pus freely; stated that he had discharged an immense quantity of purulent matter by vomiting. Troubled with copious night-sweats, preceded by slight febrile exacerbation. In three weeks more he was again *working at his trade.* The disease, however, was not eradicated.

On the 27th May medical assistance was again demanded, when he was found in a worse condition than before; very feeble and emaciated, with the liver so much enlarged as to fill nearly two-thirds of the abdomen, obstructing, materially, the operation of the medicine. There was a painful tumour above the umbilicus, and one below, to the right side, or in the right lumbar region, both of which indicated the incipient formation of abscesses. In spite of a rigid antiphlogistic treatment and every effort to arrest the formation of pus, it was evident on the 1st of June, three days after, that this had taken place. Next day upon examining the alvine dejections, it was discovered that they consisted almost exclusively of green pus. The points of the tumours were sunk, flaccid and without any fluctuation. In a few days the tumors, or abscesses were again full and doughy. At the solicitation of the family, and by the advice of Dr. Tatman, and my friend, Dr. Geo. Hill, of Opelousas, an opening was made through the abdominal parietes and a quantity of fœtid gas rushed out, allowing the abdomen to sink down into a flaccid condition. In a few days pus was discharged at the incision in considerable quantities; the orifice had no disposition to close, although the lips were brought in apposition by adhesive strips. Although he discharged pus by expectoration and per anum, yet this was the greatest outlet, and in the course of two weeks the matter ceased entirely to pass by either of the openings, notwithstanding his body was placed in several different postures. The quantity of pus at this time was small; the opening was healthy, and there was such a favorable general condition of the patient, that hopes were entertained of a final recovery. At this stage of the case his attendant, Dr. Murph, was taken very ill, and saw him no more.

From exposure, imprudence and neglect, as I am credibly informed by Dr. Tatman, and also the too free use of mercury, after the illness of his physician, a deplorable condition was brought on. Severe salivation, gangrene of the external orifice, general prostration, and wreck of the whole system supervened, and he died about the last of July, 1843, in a most loathsome condition. Dr. T. was called to see him, but it was too late—he was moribund when he saw him. I am extremely sorry to say, that owing to the peculiarly unpleasant situation of the cadaver, and the great press of other cases, there was not a post-mortem examination. And, in fact, in this particular the physicians of the South and West are culpably negligent in prosecuting these examinations. I hope, however, that a change is taking place, both in the medical gentlemen and the community, as regards autopsies.

On reviewing this remarkable case, there are many thoughts suggested to the mind, and many points of inquiry. Is it likely that he would have recovered from the first severe attack, if medical assistance had not been employed? My own impression is that he would not. The remedies employed to revive him, and the great mental adjuvant, of faith in his physician, brought him through.

It is singular that an inflammation of the liver should have occurred at that season of the year, and more especially of last year, when the winter was so very cold and protracted. Is it likely that there was a slow, chronic lesion transpiring in that organ the cause of the pain in the right hypochondrium which was increased by hard labor?

I am of opinion that there were, in truth, *four* different points of exit to the pus; for on the 27th March he was found expectorating pus, and had vomited a great quantity; here then were two. Subsequently he discharged green pus per anum, and then the external opening was made. It may be contended that the pus which was discharged by vomiting and per anum entered the alimentary canal at the same point. If it entered the stomach or duodenum, it could scarcely have passed the entire length of the intestinal tube in such an unmixed and pure condition as it was found to be; and if it entered at any point below the duodenum, could it have been discharged by emesis? My opinion is, that it was discharged into the stomach, and also into the colon.

A case somewhat similar, except that it was more rapid in its progress, is to be found in Dr. James Johnson's treatise on the diseases of Tropical Climates. On post-obit search, he says, "the liver was found one entire mass of suppuration and disease. I passed my hand from it into the stomach, to which it adhered, and through which an abscess had burst. Another adhesion had formed between the liver and the transverse arch of the colon, through which was an exit also for the matter."

A similar case, with a better result, occurred in the practice of Dr. Colledge, at Macao, China, recorded in Dr. Bell's Eclectic Journal of Medicine, for January, 1839. He first discharged the pus through the colon downwards, and afterwards through the lungs. He finally recovered.

I am inclined to think that Mr. Bundick would have recovered, if proper professional aid had been rendered after his first medical attendant was taken sick. The case should teach us never to despair, but under circumstances the most unpromising to ply our measures to the last. Few cases can occur where less is promised than was afforded by this at one time, and yet the recovery was so far completed, that the patient was able to resume his laborious trade. It is greatly to be regretted that he did not receive in his relapse the same judicious treatment which attended his first illness. The omission of the autopsy is one which I also very much regret.

March, 1844.

